# Anti-bacterial Treatment of Polyethylene by Cold Plasma for Medical Purposes

**DOI:** 10.3390/molecules17010762

**Published:** 2012-01-13

**Authors:** Anton Popelka, Igor Novák, Marián Lehocký, Ivan Chodák, Ján Sedliačik, Milada Gajtanska, Mariana Sedliačiková, Alenka Vesel, Ita Junkar, Angela Kleinová, Milena Špírková, František Bílek

**Affiliations:** 1 Polymer Institute, Slovak Academy of Sciences, Dúbravská cesta 9, 845 41 Bratislava 45, Slovakia; 2 Centre of Polymer Systems, University Institute, Tomas Bata University in Zlín, Nad Ovčírnou 3685, 760 01 Zlín, Czech Republic; 3 Faculty of Wood Sciences and Technology, Technical University in Zvolen, T.G. Masaryka 2117/24, 960 53 Zvolen, Slovakia; 4 Plasma Laboratory, Department of Surface Engineering, Jožef Stefan Institute, Jamova cesta 39, SI-1000 Ljubljana, Slovenia; 5 Institute of Macromolecular Chemistry AS CR, v. v. i, Heyrovsky Sq. 2, 162 06 Prague 6, Czech Republic; 6 Polymer Centre, Faculty of Technology, Tomas Bata University in Zlín, T.G.M Sq. 275, 762 72 Zlín, Czech Republic

**Keywords:** polyethylene, grafting, plasma treatment, immobilization, triclosan, chlorhexidine, acrylic acid

## Abstract

Polyethylene (PE) is one of the most widely used polymers in many industrial applications. Biomedical uses seem to be attractive, with increasing interest. However, PE it prone to infections and its additional surface treatment is indispensable. An increase in resistance to infections can be achieved by treating PE surfaces with substances containing antibacterial groups such as triclosan (5-Chloro-2-(2,4-dichlorophenoxy)phenol) and chlorhexidine (1,1'-Hexamethylenebis[5-(4-chlorophenyl)biguanide]). This work has examined the impact of selected antibacterial substances immobilized on low-density polyethylene (LDPE) via polyacrylic acid (PAA) grafted on LDPE by low-temperature barrier discharge plasma. This LDPE surface treatment led to inhibition of *Escherichia coli* and *Staphylococcus aureus* adhesion; the first causes intestinal disease, peritonitis, mastitis, pneumonia, septicemia, the latter is the reason for wound and urinary tract infections.

## 1. Introduction

PE is one of the most common biomedical polymers due to its excellent mechanical properties, but it suffers from insufficient biocompatibility and bioactivity [1]. PE is widely used in many biomedical applications including the production of catheters for percutaneous transluminal coronary angioplasty in medical and pharmaceutical industries [2], but infections resulting from application of this medical polymer represent the main clinical complication [3]. These infections may cause implant failure, complex revision processes and implant removal, and all can lead to patient suffering, prolonged hospitalization and even death in some cases [4]. Biocompatibility depends on many surface characteristics such as wettability [5], roughness, chemistry, surface charge, density of functional groups. The presence of hydrophobic and hydrophilic domains [6], charge [7], the functional group densities, and their conformation [8,9] play ascendant roles in affecting cell behavior [10]. Although PE has superiority concerning volume properties, its surface free energy has a low value that reflects its low wettability. This property is related to its hydrophobic and chemically inert surface without polar functional groups [11]. The solution of the problem consists in PE surface modification. Low-temperature plasma can be suggested as the appropriate procedure for the hydrophilization of the surface. Due to the plasma treatment surface the free energy is increased as a result of introduction of polar functional groups on the treated surface, thus making the surface of PE more hydrophilic [12,13].

Developing plasma techniques belong to an important class of polymer surface modification techniques where a very thin layer of the polymer surface is treated without any changes in bulk. Moreover, plasma technology is based on ecological, clean and dry processes suitable for industrial applications without the use of chemicals. Low-temperature plasma is often used in many applications, for example in the electronic, aeronautic, automotive, medical [14], biomedical, textile, optical and paper industries [15]. In this process a polymer is exposed to a plasma reactive species such as ions, electrons, excited atoms and molecules, which cleave existing chemical bonds and form new reactive functional groups, which may initiate or participate in grafting, polymerization, or cross-linking reactions on the surface. Plasma processing can significantly contribute to adhesion improvement by removing surface contamination and to surface morphology changes through increased roughness due to etching [16,17].

The Diffuse Coplanar Surface Barrier Discharge (DCSBD) plasma generator [18] appears to be an effective tool for creating macroscopically homogeneous plasmas, which has many advantages compared with conventional devices. The most important advantage considering the application of DCSBD includes performance at atmospheric pressure, which is significant in terms of continuous industrial technologies. Another advantage is that the plasma does not directly contact the electrodes, which protects these from wear [19]. DCSBD equipment consists of two parallel banded system of electrodes (usually 1-mm wide, 50 micron thick, with 0.5 mm spacing between the strips, made of Ag-paste) embedded in 96% Al_2_O_3_-promotion of natural purity. Such an arrangement of electrodes leads to a visually almost completely macroscopically homogeneous diffusion plasma [20,21,22].

Adhesion and surface growth of bacteria, also called biofilm formation, is a widespread problem [23]. To prevent its formation, anti-infection modification of polymers for medical applications may be applied. Anti-infective properties of polymers can be achieved by following: (a) anti-infection agents mixed in the polymer; (b) copolymerization anti-infection agents with monomer; (c) appropriate surface treatment of medical polymers.

**Figure 1:**
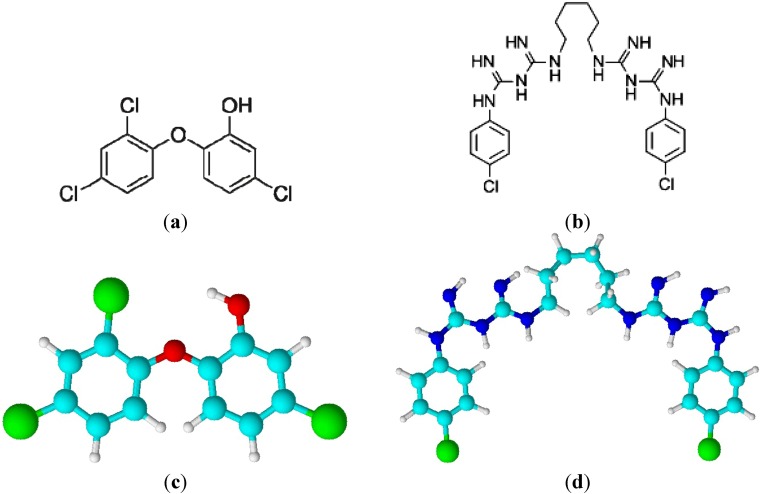
(**a**) triclosan; (**b**) chlorhexidine; (**c**) 3D structure of triclosan; (**d**) 3D structure of chlorhexidine.

Antibacterial surface modification is controlled by the physical-chemical interactions between bacteria and polymer surface. This treatment has several advantages, because it does not influence the bulk properties of the polymer, antibacterial agents are not released from the polymer volume, and the technique is relative simple and effective. Triclosan [24] and chlorhexidine [25] (Figure 1) shows straight, steady, broad-spectral antibacterial efficiency and very low clinical toxicity in clinical tests. This treatment in combination with plasma can affect significantly biochemical and physical properties of LDPE [3] by following a multistep physicochemical approach [26]. In the first step, formation of functional groups on the polymer surface is necessary via the plasma species created by a DCSBD generator [27,28,29]. 

In the second step, an end-functionalized polymer brush is formed on polymer surface via radical graft polymerization of acrylic acid (AA), which is anchored on the plasma treated surface [30]. The PAA grafted on the LDPE surface represents a new approach for subsequent antibacterial treatment. Finally, biomolecules are immobilized on this pre-treated surface using EDAC coupling, whereby carboxyl groups of AA are then activated and ready to provide the immobilization sites [31].

## 2. Results and Discussion

### 2.1. Surface Wettability

**Figure 2:**
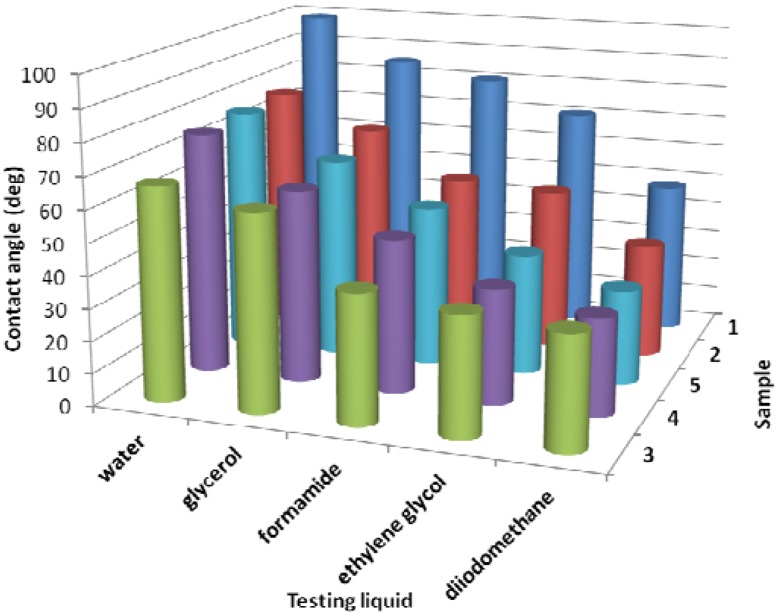
Contact angle *vs.* surface treatment and *vs.* testing liquid; 1 - untreated LDPE; 2 - plasma-treated; 3 - AA grafted; 4 - triclosan coated; 5 - chlorhexidine coated.

Wetting (wettability) can be defined as the degree to which a solid is wetted. When a drop is totally spread on solid surface and the contact angle approaches 0 deg, then the complete wettability of the surface is achieved. However, in many cases it is only a partial wettability occurs (or non-wettability). Contact angle measurements are usually used for estimating the extent to which a solid surface will be wetted. Wettability can be expressed as relative strength of cohesion (liquid/liquid) and adhesion (solid/liquid) forces. Weak cohesion with strong adhesion due to the very low contact angle is close to full wettability. If solid/liquid interactions decrease and liquid/liquid interactions increase, wettability decreases. If the contact angle of water is 90 deg or more the polymeric surface is hydrophobic. This relates with poor wettability, low surface energy and weak adhesion of the polymer. On the other side drop with a small contact angle relates to more hydrophilic surface, that causes better wettability, adhesion and higher surface energy of investigated material. The contact angles changes of testing liquid set, graft yield (GY), and surface free energy (γ^tot^) and its components of antibacterial treated LDPE are shown in Table 1. The graft yield (GY) was calculated by the following equation: 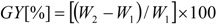, where W_1_ and W_2_ represent the weight of the samples before and after surface treatment [32]. The graphic changes of contact angles of testing liquids caused by antibacterial treatment are shown in Figure 2. The water contact angle (θ_w_) of untreated LDPE (Sample 1) achieves the highest values from the all samples because it is polymer with hydrophobic and chemical inert surface. θ_w_ significantly decreased after plasma effect of the Sample 2 when different functional groups were introduced on to the surface formed from plasma species and therefore the treated surface acquired more polar or hydrophilic character. The highest decrease of the contact angle was observed in case of surface covered by polyacrylic acid (PAA, Sample 3) which corresponds to its hydrophilic character. Also triclosan (Sample 4) and chlorhexidine (Sample 5) immobilization led to θ_w_ decrease. For investigation of other physicochemical parameters of the treated surface Lifshitz-Van der Waals/acid-base (LW/AB) theory was used, which allows to obtain γ^tot^ and its components such as non-polar LW (γ^LW^) and polar AB (γ^AB^) components. LW indicates the total dispersive Lifshitz-Van der Waals interaction and AB refers to the acid-base or electron-acceptor/electron donor interaction according to Lewis [33]. LDPE belongs to group of low-energy polymeric materials and therefore γ^tot^ of Sample 1 achieves very low values which correspond with difficulties during processing, such as dyeing, printing and bonding (low adhesion). This can be removed by plasma treatment of LDPE when γ^tot^ can significantly increases as in the case of Sample 2. The largest increase of γ^tot^ and γ^AB^ was observed for Sample 3 due to highest polarity in comparison with other samples as a result of polar oxygen group’s presence. Sample 4 and 5 showed similar increases of surface free energy values, thereby confirming the increase in wettability.

**Table 1:** Surface properties of LDPE treated by multistep process.

Sample	*θ_w_* * (°)*	*θ_e_* * (°)*	*θ_g_* * (°)*	*θ_d_* * (°)*	*θ_f_* * (°)*	*γ^−^ (mN/m)*	*γ^+^ (mN/m)*	*γ^AB^* * (mN/m)*	*γ^LW^* * (mN/m)*	*γ^tot^* * (mN/m)*	*GY* *(%)*
1	99.2 (±0.6)	70.9 (±1.2)	85.3 (±0.9)	48.4 (±1.2)	80.7 (±0.9)	1.0	0.1	0.7	34.5	35.2	-
2	77.5 (±1.1)	51.0 (±2.8)	67.1 (±2.8)	36.0 (±1.2)	52.8 (±1.5)	6.6	0.1	1.1	41.4	42.6	0.0
3	66.9 (±0.7)	32.1 (±2.4)	57.2 (±2.7)	32.5 (±1.6)	37.0 (±2.0)	10.4	0.5	4.5	43.7	48.1	0.5
4	75.8 (±1.6)	36.1 (±0.7)	60.4 (±1.0)	30.5 (±1.5)	48.3 (±1.2)	5.0	0.4	2.8	44.0	46.8	1.8
5	76.7 (±0.5)	38.1 (±2.5)	63.2 (±2.72)	30.0 (±1.6)	50.4 (±1.5)	5.2	0.2	2.0	44.4	46.4	2.0

w = deionized water, e = ethylene glycol, g = glycerol, d = diiodomethane, f = formamide; * Sample 1: untreated LDPE; Sample 2: plasma-treated; Sample 3: AA grafted; Sample 4: triclosan coated; Sample 5: chlorhexidine coated.

### 2.2. Adhesive Properties

**Figure 3:**
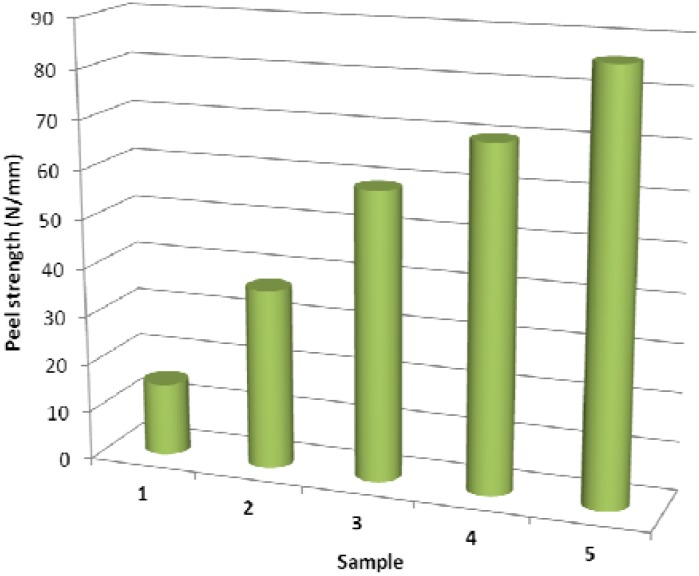
Peel strength *vs.* surface treatment; 1 - untreated LDPE; 2 - plasma-treated; 3 - AA grafted; 4 - triclosan coated; 5 - chlorhexidine coated.

The results of peel strength measurements of adhesive joint to poly(acrylate) are shown in Figure 3. Surface free energy changes are closely related to adhesion between two materials in contact. Therefore, the increased wettability resulted in an increase of adhesion strength of adhesive joint to more polar poly(acrylate). However, adhesion depends not only chemical composition and the chemical nature of the surface, but also on surface morphology (roughness). The rougher is the surface the higher is the adhesion and *vice versa*. Thus, adhesion is a complex parameter consisting of several related chemical and physicochemical properties. Therefore, in the case of Sample 3 even though the surface energy reaches its highest value the peel strength is less than for Sample 4 and 5. Cross-linking occurred in Sample 5 (via glutaraldehyde) is another factor that contributes to the increase in the adhesion strength [34].

### 2.3. Surface Morphology

**Figure 4:**
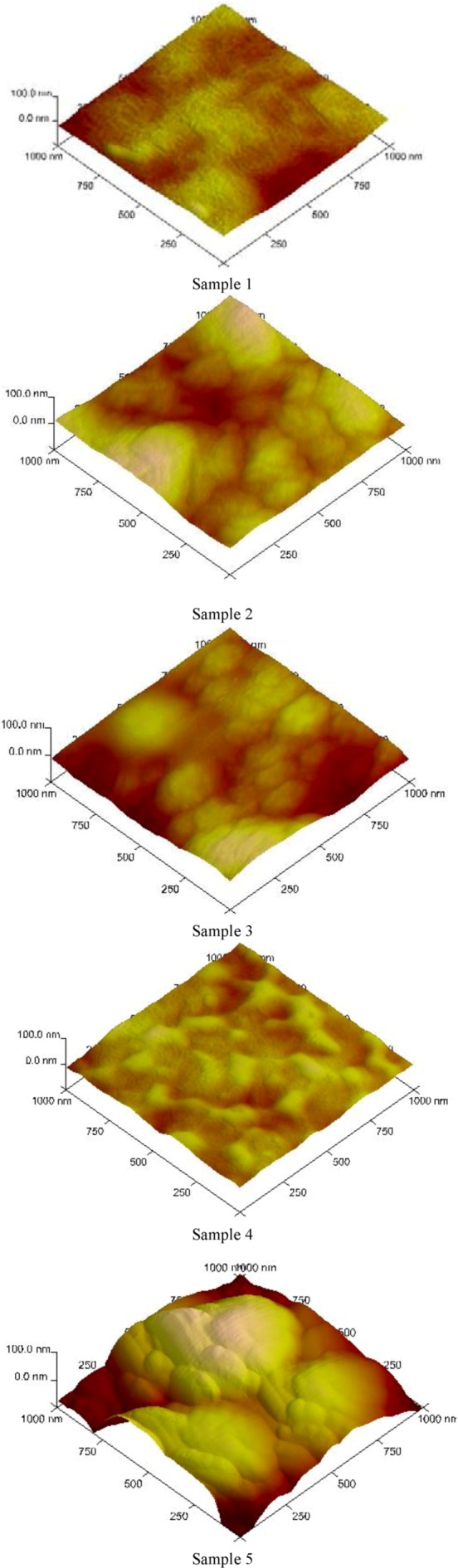
AFM surface changes for Sample 1–5: 1 - untreated LDPE; 2 - plasma-treated; 3 - AA grafted; 4 - triclosan coated; 5 - chlorhexidine coated.

Surface morphology changes (according to AFM measurements) of antibacterial treated LDPE by multistep process via DCSBD are shown in Figure 4. The relief of Sample 1 is only slightly wavy, caused by inequalities in the production of LDPE foils. The plasma effect led to the slightly increase of LDPE surface roughness as a result of surface changes by re-organization of the surface microstructure by chemical (functionalization) and mechanical (ablation) processes. The plasma grafting of LDPE by acrylic acid results in the creation of a brush-like pattern appropriate for subsequent modification. Triclosan coating alteration of the surface topography led to characteristic textures. The cross-linking agent (glutaraldehyde) was used to improved chlorhexidine binding to LDPE resulting in the formation of cross-linked structure and therefore the significant changes in the surface morphology and roughness were observed.

### 2.4. Surface Chemistry

#### 2.4.1. Analysis of FT-IR-ATR Spectra

The FT-IR-ATR measurements provide mostly semi-quantitative information on the chemical changes of the near-surface region, because the measured thickness of the layer is limited to 4 μm for ZnSe crystal. The Ge crystal has by far the highest refractive index of all the ATR materials available which means that the effective depth of penetration is lower than in case of ZnSe [35]. For better visualization the infrared spectra of virgin LDPE and modified material together with the pure triclosan were split into three different wavenumber regions. The spectrum of the untreated LDPE is a typical polyethylene spectrum with a small number of characteristic peaks. After air plasma exposure of the pristine material, the characteristic oxygen functional groups were introduced and therefore significant changes in the measured spectrum have been observed. These changes seem to be caused by the incorporation of some hydroxy or peroxy groups after the plasma treatment of LDPE in air (evidence for that statement is the appearance of two broad peaks between 3,600–3,050 cm^−1^ and 1,800–1,520 cm^−1^, respectively). Carbonyl stretching is one of the easiest absorptions to recognize in an infrared spectrum. It is usually the very intense band in the spectrum. In this spectrum also the appearance of two smaller peaks at 1,280 cm^−1^ and 1,120 cm^−1^ are seen. 

**Figure 5:**
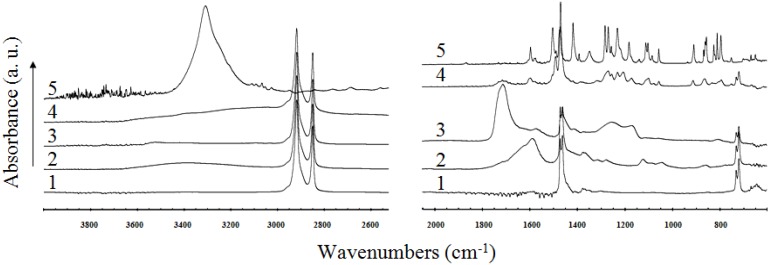
FT-IR-ATR spectra of: 1 - untreated LDPE; 2 - plasma treated; 3 - AA grafted; 4 - triclosan coated; 5 - pure triclosan.

Significant changes in the spectra are also observed in (both) cases of LDPE-PAA grafting and after the subsequent triclosan coating. In the spectrum of grafted material one can observe some characteristic peaks of polyacrylic acid, *i.e.*, the most intense peak at 1,712 cm^−1^ (carbonyl band, C=O stretching) and also some unresolved peaks in the fingerprint region (1,300–1,100 cm^−1^, C-O stretching and CH_2_ bending). After triclosan treatment the shape of the spectrum changes, as can be seen in Figure 5. These changes are significant almost in a whole mid-infrared region, especially in the region below 1,700 cm^−1^. Because of the simple spectrum of LDPE (small numbers of peaks) in comparison with the spectrum of triclosan, it is assumed that almost all changes in the spectrum of the triclosan coated LDPE are originated due to addition of triclosan. The presence of triclosan in the treated sample is confirmed by an appearance of a number of peaks, which are also present in the spectrum of the pure triclosan (e.g., at 1,491 cm^−1^, benzene ring vibration), and also without doubt by a peak at 752 cm^−1^ (stretching mode of C-Cl in the triclosan molecule). The shifts in the maxima of individual peaks and the changes in their shapes can be probably assigned to bonding of triclosan molecules to LDPE surface and inhibition of their unrestricted motion.

**Figure 6:**
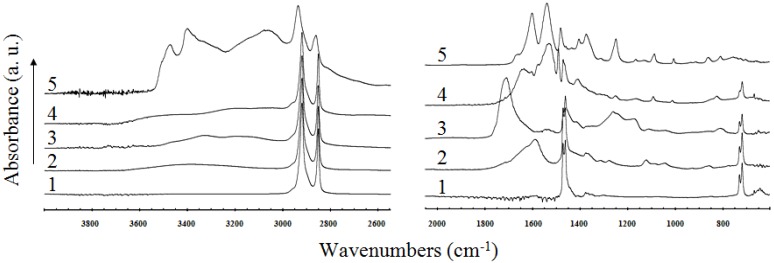
FT-IR-ATR spectra of: 1 - untreated LDPE; 2 - plasma treated; 3 - AA grafted; 4 - chlorhexidine coated; 5 - pure chlorhexidine.

More significant changes in spectra are observed in (both) cases of LDPE-PAA grafting and also after subsequent chlorhexidine coating. After chlorhexidine treatment the shape of the spectrum changes, as seen in Figure 6. These changes are significant almost in the whole mid-infrared region, especially in the region below 1,700 cm^−1^. The presence of chlorhexidine is confirmed by the appearance of a peak at 1,640 cm^−1^ (C=N vibration) and also undoubtedly by a peak at 1,530 cm^−1^ (stretching mode of aromatic ring in the chlorhexidine molecule).

#### 2.4.2. Analysis of XPS Spectra

LDPE samples with different coatings were analyzed by the XPS method. The purpose of these analyzes was to prove the presence of the coating on LDPE samples which were previously treated in air plasma and grafted with acrylic acid. For each sample the surface composition was measured at two different spots on the surface. This allowed calculation of the average surface composition, which is shown in Table 2.

**Table 2:** Average surface composition of the LDPE samples as revealed by XPS.

Sample	C1s	N1s	O1s	Na1s	Cl2p	S2p
1	100	0	0			
2	76.3	4.0	19.8			
3	84.1	/	15.6			0.4
4	89.1	2.0	8.4	0.4	0.2	
5	86.8	6.7	5.0		1.5	

* Sample 1: untreated LDPE; Sample 2: plasma-treated; Sample 3: AA grafted; Sample 4: triclosan coated; Sample 5: chlorhexidine coated.

**Figure 7:**
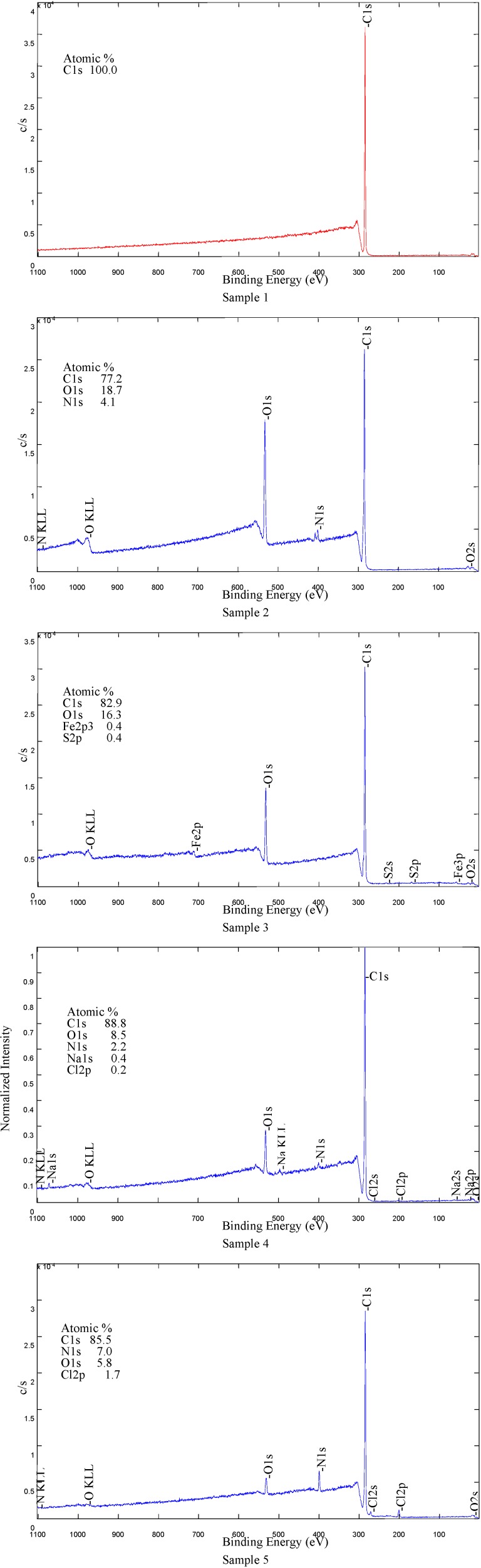
XPS survey-scan spectra of Samples 1–5 with atomic compositions; Sample 1 - untreated LDPE; Sample 2 - plasma-treated; Sample 3 - AA grafted; Sample 4 - triclosan coated; Sample 5 - chlorhexidine coated.

**Figure 8:**
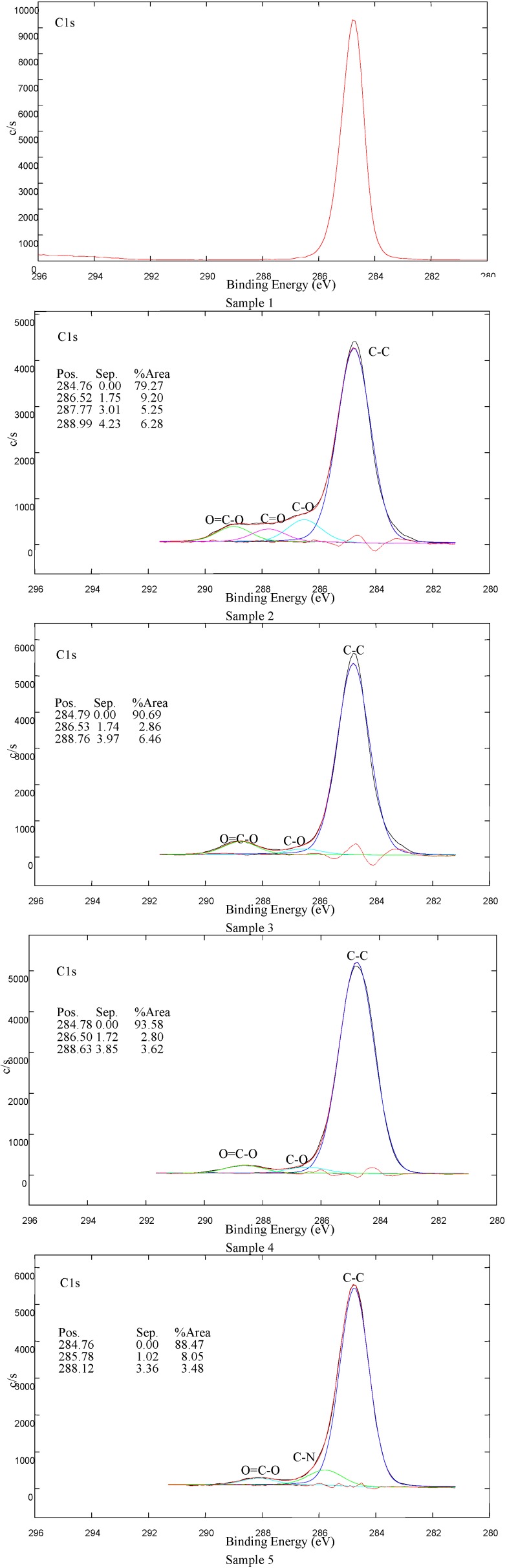
Carbon C1s peaks of Sample 1–5; Sample 1 - untreated LDPE; Sample 2 - plasma-treated; Sample 3 - AA grafted, Sample 4 - triclosan coated; Sample 5 - chlorhexidine coated.

**Figure 9:**
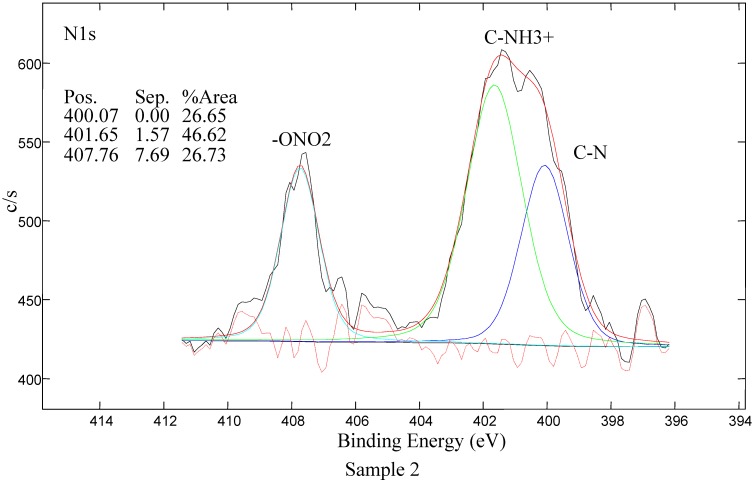
Nitrogen N1s peak for Sample 2 - plasma-treated.

XPS survey-scan spectra of Samples 1–5 are shown in Figure 7 and the carbon C1s peaks of Samples 1–5 are shown in Figure 8. Moreover the nitrogen N1s peak for Sample 2 is shown in Figure 9. For LDPE treated in air plasma different oxygen functional groups and also some nitrogen groups were found. Sample 3 shows mostly the presence of carboxyl groups. For this sample also some traces of iron, about 0.4 at %, were detected. In case of air plasma treatment the peak could include carboxyl as well as ester groups, which could not be resolved with XPS analysis. In the case of AA grafting we believe that this peak presents only carboxylic groups which originate from AA. Furthermore air plasma treatment results also in incorporation of other oxygen functional groups, such as carbonyl and hydroxyl, which can be clearly seen from Figure 8. Comparison of carbon C1s peaks of Sample 1 and Sample 2 are shown in Figure 8. As expected, the carbon spectrum of an untreated sample has only one peak due to C-C bonds. After plasma treatment, new peaks due to different carbon-oxygen bonds appear. As seen in Table 2, some nitrogen is found as well but the C-N peak is overlapping with the C-O peak. Therefore, this peak does not appear in Figure 8 for C1s peak of Sample 2. Nitrogen N1s peak of Sample 2 is composed from different modes of chemical binding of nitrogen atoms. More details about nitrogen binding can be seen in Figure 9 for N1s of Sample 2. As immobilization of biomolecules is done by carboxylic groups, grafting of AA to plasma treated LDPE is important to obtain only carboxylic groups on the polymeric surface. Carbon C1s peak of LDPE sample grafted with acrylic acid (AA) is present in Figure 8. As expected carboxyl group due to AA is detected at the surface. For Sample 4 mostly oxygen and some nitrogen (which is not from triclosan) were found. Concentration of Cl is very low. The coating is probably very thin, since the carboxylic group, which is clearly seen in spectrum, originates from AA, which is below the triclosan. Peak due to C-O/C-OH bond is associated with the presence of triclosan. For Sample 5, nitrogen and chlorine originating from the triclosan coating were detected. See also Figure 8 showing C-N bonds from chlorhexidine coating and carboxylic part from AA.

#### 2.4.3. Antibacterial Activity Assessment

Table 3 shows inhibition zone area results. The inhibition zone area was calculated as the sample surface area deducted from the total area of the inhibition zone. The results show that untreated (Sample 1), plasma treated (Sample 2) as well as acrylic-acid grafted sample (Sample 3) do not display any antibacterial activity against both *Escherichia coli* and *Staphylococcus aureus* strains. The sample coated with triclosan (Sample 4) does meet the expected antibacterial requirements. The average inhibition zone for the Gram-negative *Escherichia coli* strain is of 115.1 mm^2^ and for the Gram-positive *Staphylococcus aureus* 493.1 mm^2^. These values prove the antibacterial activity of the prepared layers as well as confirm XPS measurements. Similar results were obtained for chlorhexidine coated samples (Sample5). The average inhibition zone value of 42.2 mm^2^ was calculated for *Escherichia coli* and 288.1 mm^2^ for *Staphylococcus aureus* strain. It is worth mentioning, that both antibacterial agents are more active against Gram-positive bacteria. Finally, triclosan coated samples show better results among the two antibacterial substances used.

**Table 3:** Inhibition zone area measurement.

LDPE	Inhibition zone (mm^2^)			Average value (mm^2^)
	1	2	3	
*Escherichia coli*				
Sample 1	0	0	0	0
Sample 2	0	0	0	0
Sample 3	0	0	0	0
Sample 4	105.8	118.3	121.2	115.1
Sample 5	40.2	43.8	42.5	42.2
*Staphylococcus aureus*				
Sample 1	0	0	0	0
Sample 2	0	0	0	0
Sample 3	0	0	0	0
Sample 4	475.0	496.3	507.9	493.1
Sample 5	286.4	279.3	298.5	288.1

* Sample 1: untreated LDPE; Sample 2: plasma-treated; Sample 3: AA grafted; Sample 4: triclosan coated; Sample 5: chlorhexidine coated.

## 3. Experimental

### 3.1. Materials

LDPE BRALEN FB 2-17 foils: Slovnaft MOL (Slovakia), containing no processing additives, the thickness of LDPE film was 20 μm, density = 0.918 g·cm^−3^, mass flow rate (MFR at 190 °C, 2.16 kg) = 2 g per 10 min, Vicat softening temperature = 96 °C. This type of LDPE is suitable for food contact. The product complies with Food Contact Regulations and the grade is suitable for manufacturing of pharmaceutical packing-products.

Triclosan (5-Chloro-2-(2,4-dichlorophenoxy)phenol): Irgasan, C_12_H_7_Cl_3_O_2_, Fluka Analytical (Italy), white powder, Assay ≥ 97.0% (HPLC), M_r_ = 289.54 g·mol^−1^, ash ≤ 0.1%, melting point = 56–58 °C.

Chlorhexidine (1,1'-Hexamethylenebis[5-(4-chlorophenyl)biguanide]): imidodicarbonimidic diamide, C_22_H_30_Cl_2_N_10_, Aldrich Chemistry (Spain), white powder, Assay = 98%, M_r_ = 505.46 g·mol^−1^, melting point = 134 °C.

Acrylic acid (Prop-2-enoic acid): C_3_H_4_O_2_, colorless liquid, Acros Organics (Belgium), Assay = 99.5%, extra pure, stabilized with 180 to 220 pm monomethyl ether of hydroquinone (MEHQ), M_r_ = 72.06 g·mol^−1^, flash point = 48 °C, density = 1.050 g·cm^−3^, boiling point = 139 °C.

EDAC (N-(3-dimethylaminopropyl)-N'-ethylcarbodiimide hydrochloride): C_8_H_17_N_3_·HCl, Fluka (USA), purum, Assay = 98.0%, M_r_ = 191.70 g·mol^−1^, melting point = 110–115 °C.

Glutaraldehyde (Pentane-1,5-dial): C_5_H_8_O_2_, clear liquid, was used as 25.0 wt% aq. solution, M_r_ = 100.12 g·mol^−1^, density = 1.06 g·cm^−3^, melting point = −14 °C, boiling point 187 °C.

Ethylene glycol (Ethane-1,2-diol): C_2_H_6_O_2_, Sigma-Aldrich (USA), anhydrous, Assay = 99.8%, M_r_ = 62.07 g·mol^−1^, flash point = 111 °C, melting point = −13 °C, boiling point = 195–197 °C.

Glycerol (Propane-1,2,3-triol): C_3_H_8_O_3_, Sigma (Germany), for molecular biology, Assay = 99%, M_r_ = 92.09 g·mol^−1^, density = 1.262 g·cm^−3^, melting point = 20 °C, flash point = 160 °C, boiling point = 182 °C/20 mmHg. 

Formamide (Methanamide): CH_3_NO, Sigma (USA), deionized, Assay = 99.5%, M_r_ = 45.04 g·mol^−1^, density = 1.132 g·cm^−3^, melting point = 2 °C, flash point 150 °C, boiling point = 210 °C/760 mmHg.

Diiodomethane: CH_2_I_2_, colorless liquid with chloroform-like odour, Assay = 99%, Reagent Plus, Sigma-Aldrich (Germany), containing copper as stabilizer, M_r_ = 267.84 g·mol^−1^, density = 3.325 g·cm^−3^, melting point = 5–8 °C, flash point = 110 °C, boiling point = 67–69 °C.

Dichloromethane: CH_2_Cl_2_, mikroCHEM (SVK), Assay = 99.5%, M_r_ = 84.93 g·mol^−1^, density = 1.33 g·cm^−3^, melting point = −96.7 °C, boiling point = 39.6 °C. 

### 3.2. Plasma Treatment

**Scheme 1:**
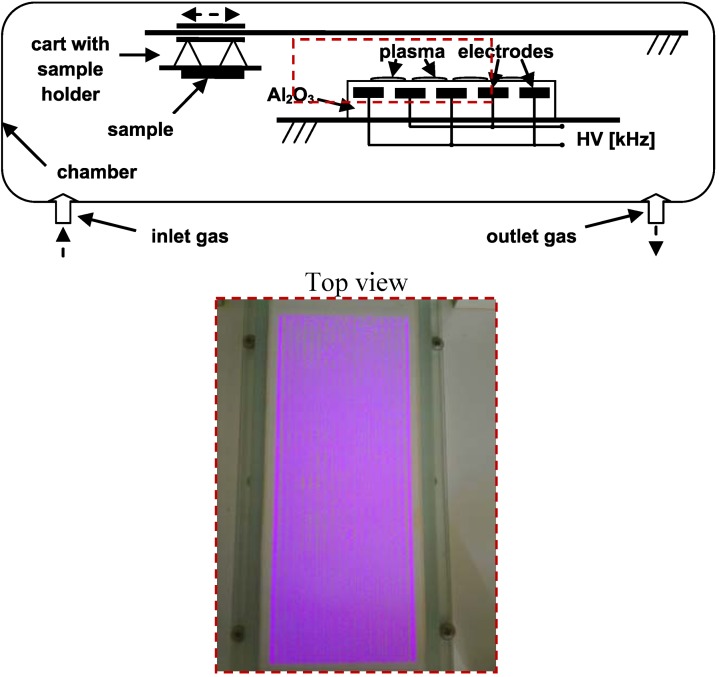
DCSBD scheme and detail of burning plasma panel.

The LDPE foils were first cleaned with dichloromethane to remove impurities. Then the LDPE foil activation was carried out under dynamic conditions at atmospheric pressure and room temperature with the DCSBD equipment developed at Comenius University (Department of Experimental Physics, Faculty of Mathematics, Physics and Informatics) in Bratislava. The schematic representation with description of this system is given in Scheme 1. The treatment was performed with the following settings: power supply = 200 W, plasma treatment time = 15 s, in air atmosphere and all samples were treated on both sides. DCSBD equipment generates macroscopically homogeneous plasma without direct contact with the electrodes, which protects the electrodes from wear. Plasma is generated by two parallel banded system of electrodes (1-mm wide, 50 micron thick, with 0.5 mm spacing between the strips, made of Ag-paste) embedded in 96% Al_2_O_3_-promotion of national purity, while the electrodes are supplied via high frequency sinusoidal voltage (~15 kHz, Um~10 kV). Such an arrangement of electrodes and supply voltage leads to visually almost perfectly homogeneous diffusion plasma.

### 3.3. Grafting by PAA

Immediately after plasma treatment the LDPE foil was immersed into 10 volume % aqueous solution of AA for 24 h at 30 °C in order to initiate of radical graft polymerization of AA onto activated surface of LDPE foil. This solution contained also 0.1 wt.% sodium metabisulfite as a relevant reductant to inhibit AA homopolymerization. After AA polymerization PAA brushes were created onto LDPE surface that are suitable for binding antibacterial agents. After removing the samples from the solution the grafted foils were washed in deionized water for 5 min at 30 °C in an ultrasonic bath for removal weakly bound PAA and unreacted AA species on the surface LDPE.

### 3.4. Antibacterial Immobilization

**Scheme 2:**
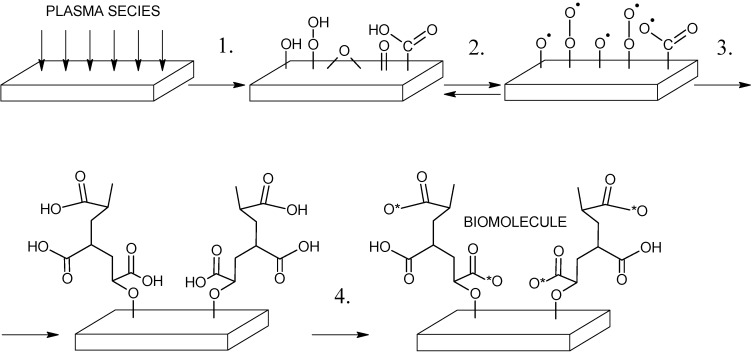
Multistep approach of bimolecular binding: 1. plasma treatment; 2. radical generation; 3. AA radical graft polymerization; and 4. antibacterial deposition.

LDPE grafted by PAA was immersed at 4 °C for 6 hours into 0.1 w/v% aqueous solution of EDAC that acts as an activator of carboxyl groups where *O*-acylisourea is produced and it has possibility to react with reducing agents. The sample pre-prepared by such way was then immersed into solution of triclosan and chlorhexidine. The first solution was prepared as 2 w/v% solution of triclosan in absolute ethanol and the latter as 2 w/v% solution of chlorhexidine in 70 v/v% isopropanol aqueous solution for 24 h at 30 °C in an oven. Moreover the coated LDPE by chlorhexidine was then yet immersed into 1 w/v% aqueous solution of glutaraldehyde overnight at 4 °C to better immobilization of chlorhexidine onto the surface via cross-linking. The antibacterial treated samples were thoroughly washed and then dried for 24 h at room temperature to constant weight. The mechanism of antibacterial treatment is described in Scheme 2.

### 3.5. Surface Wettability Evaluation

The wettability of LDPE treated by multistep process via PAA plasma grafted and antibacterial immobilization were carried out by the measurement of contact angle using sessile drop technique using Surface Energy Evaluation system (SEE system with CCD camera, Advex Instruments, made in Czech Republic). This system contains sensitive CCD camera with the highest resolution equal to 1,280 × 960 due to high screen capture. Contact angle was measured by placing a small drop of testing liquid on a surface treated LDPE. The angle formed between the solid/liquid interface and the liquid/vapor interface is referred to as the contact angle. Deionized water, ethylene glycol, glycerol, formamide, diiodomethane were used as testing liquids, applied volume was 3 μL (elimination of influence of gravity) and a static contact angle was measured shortly after the drop formation when a thermodynamic equilibrium is reached between the three phases: solid, liquid, and gas. Surface energy (γ^tot^), its polar acid-base (γ^AB^), dispersive (γ^LW^), electron-acceptor (γ^−^) and electron-donor (γ^+^) components were calculated by Acid-Base regression model using method of least squares.

### 3.6. Adhesive Properties Assessment

The adhesive properties, namely peel strength (force per unit width) of the adhesive joint of antibacterial treated LDPE by triclosan and chlorhexidine via DCSBD to poly(2-ethylhexyl acrylate) deposited onto polypropylene foil (with 15 mm width), were carried out by measurements of 90° peel test at a 10 mm per minute rate of peel using a 100 N universal INSTRON 4301 dynamometer (England). Ends of the polymer film were firmly fixed in the jaws of dynamometer so that tension was evenly distributed across the entire width of the surface.

### 3.7. Surface Topography Analysis

The surface morphology and local surface heterogeneities of the modified polymer were measured by AFM. All measurements were performed under ambient conditions using a commercial atomic force microscope (NanoScopeTM Dimension IIIa, MultiMode Digital Instr., USA) equipped with a PPP-NCLR tapping-mode probe (Nanosensors^TM^, Switzerland; spring constant 39 N·m^−1^, resonance frequency ≈ 160 kHz). The surface properties of all the films were measured in x and y axis sizes between 2 to 25 μm on different sites of the films in order to find characteristic and significant surface features. The AFM analyses were performed in tapping mode for all the images. This technique allows the obtaining either two- or three-dimensional information of both height and material heterogeneity contrast with high resolution when recording height and phase shifts simultaneously.

### 3.8. Surface Chemistry Investigation

#### 3.8.1. XPS

Samples were analyzed with a TFA XPS Physical Electronics XPS instrument. The base pressure in the chamber was about 6 × 10^−8^ Pa. The samples were excited with X-rays over a 400 µm spot area with a monochromatic Al K_α1,2_ radiation at 1,486.6 eV. The photoelectrons were detected with a hemispherical analyzer positioned at an angle of 45° with respect to the normal to the sample surface. Survey-scan spectra were made at a pass energy of 187.85 eV and 0.4 eV energy step. An electron gun was used for surface neutralization. The concentration of elements was determined by using MultiPak v7.3.1 software from Physical Electronics, which is supplied by the spectrometer producer.

#### 3.8.2. FT-IR-ATR

Attenuated total reflectance FTIR measurements were performed on a NICOLET 8700 FTIR spectrometer (Thermo Scientific) through the single bounce ATR accessory equipped with Ge crystal at an angle of incidence 45°. For each measurement the spectral resolution and the number of scans were 2 cm^−1^ and 64, respectively. The quality of spectra depends on good contact between the crystal and the sample. This requirement was achieved through the use of a pressure clamp. The acquired spectra were analyzed using spectroscopic software OMNIC™, v. 8.1.

### 3.9. *In Vitro* Antibacterial Test

Bacterial adhesion and biofilm experiments were performed using Gram-positive (*S. aureus* 3953) and Gram-negative (*E. coli* 3954) bacteria. Circular shaped specimens (d ≈ 8 mm) were cut from pristine and modified LDPE samples. A so called agar diffusion plate (inhibition) test was performed for antibacterial activity evaluation of tested substrates. The polymer samples were washed in ethanol and dried under laboratory conditions. The substrates prepared by such a way were placed on agar plate (Nutrient Agar No. 2 M1269, Hi Media Laboratories Pvt. Ltd.) inoculated by bacterial suspension. The bacterial suspension volume was 100 µL for all samples. Bacteria concentration was 10^7^ units·mL^−1^ and incubation time was 24 h at the temperature 37 °C. After that, inhibition zone diameter was measured in 5 directions and average value was calculated. Each test was repeated in triplicate.

## 4. Conclusions

This work was aimed at examining the impact of selected antibacterial agents, namely triclosan and chlorhexidine bound to the surface of LDPE. DCSBD plasma treatment leads to increased surface free energy, roughness and surface wettability by introducing characteristic oxygen groups. A DCSBD plasma generator was used as activator of the LDPE surface for efficient binding of acrylic acid and for its transformation to polymeric form by radical polymerization. Thus the bound acrylic acid created polymer brushes on the polymer surface that provided physical forces to bind antibacterial agents in an effective manner. The presence of triclosan and chlorhexidine was confirmed by different surface analysis techniques. Moreover the antibacterial effect of such treated LDPE film was proven by *in vitro* bacterial tests against *E. coli* and *S. aureus* when adhesion of bacteria to polymer was effective diminished.
